# Supplementary material for: Probing relaxed myosin states in hypertrophic cardiomyopathy by second harmonic-generation microscopy
Source: bioRxiv. 2025 May 13:2025.05.08.652664. Preprint. [Version 1] doi: 10.1101/2025.05.08.652664 (PMC12132358; doi:10.1101/2025.05.08.652664)
Supplement: Supplement 1 [file media-1.pdf]

SUPPLEMENTARY FIGURES

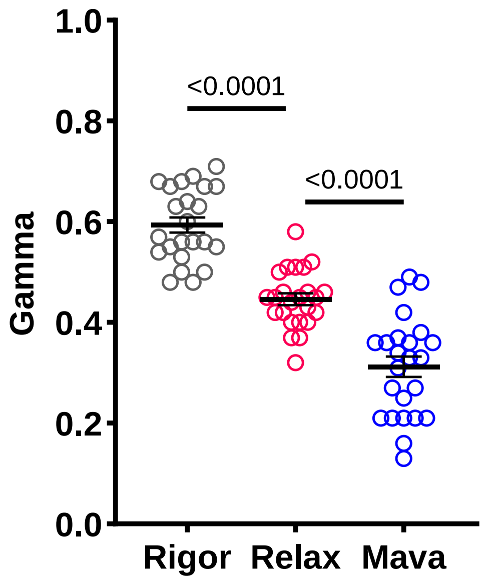

Figure S1: Rabbit psoas myofibrils. Graph illustrating γ values among the Rigor state, Relax state, and after exposure of Mavacamten (Mava), in rabbit skinned psoas myofibrils. Data are reported as mean ± S.E.M. A one-way repeated measures ANOVA was performed with a Tukey post-hoc correction.

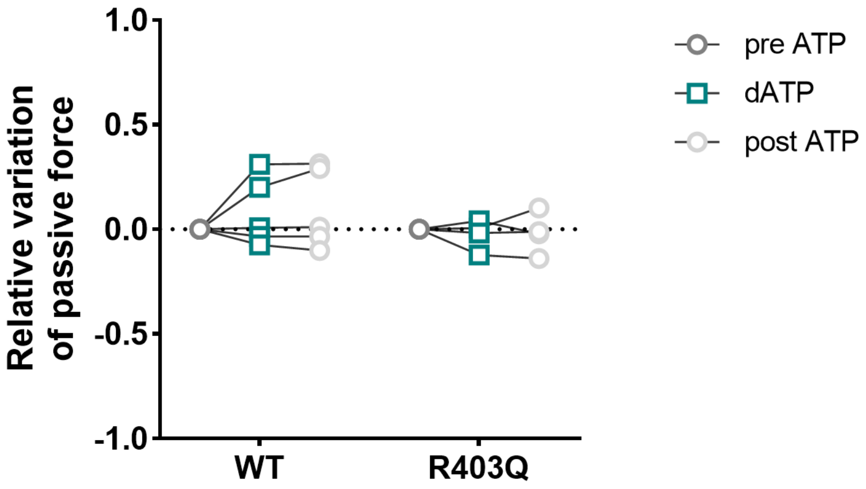

Figure S2: Passive force measurements of myofibrils. The graph shows passive force variation recorded in myofibrils initially in ATP-containing solution (pre ATP), followed by exposure to 100% 2-deoxyATP solution (dATP), and subsequently returned to ATP-containing solution (post ATP). Repeated measures were performed on the same myofibrils to assess changes in passive force under matched conditions.

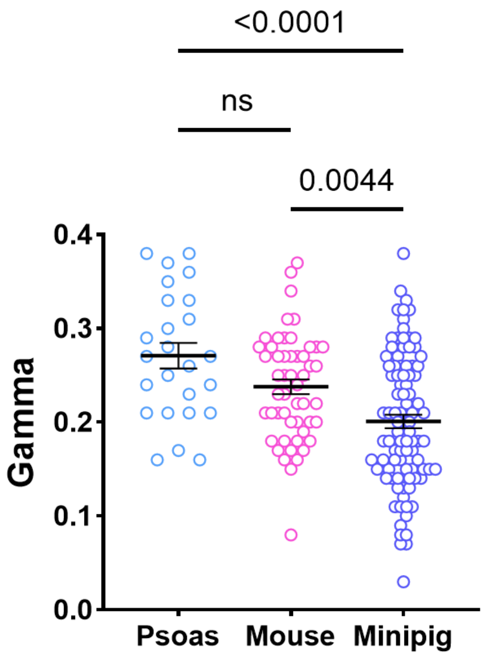

Figure S3: Comparison of γ values in demembranated preparations, from different species, exposed to Mavacamten. The graph shows γ values obtained from rabbit psoas, mouse cardiac, and minipig cardiac muscle preparations, highlighting differences under the effect of Mavacamten. Data are reported as mean ± S.E.M. A one-way repeated measures ANOVA was performed with a Tukey post-hoc correction.
